# MEPP: more transparent motif enrichment by profiling positional correlations

**DOI:** 10.1093/nargab/lqac075

**Published:** 2022-10-17

**Authors:** Nathaniel P Delos Santos, Sascha Duttke, Sven Heinz, Christopher Benner

**Affiliations:** Department of Biomedical Informatics, University of California San Diego, 9500 Gilman Drive, La Jolla, CA 92093-0634, USA; School of Molecular Biosciences, College of Veterinary Medicine, Washington State University, Pullman, WA, USA; Department of Medicine, University of California San Diego, 9500 Gilman Drive, La Jolla, CA 92093-0634, USA; Department of Medicine, University of California San Diego, 9500 Gilman Drive, La Jolla, CA 92093-0634, USA

## Abstract

Score-based motif enrichment analysis (MEA) is typically applied to regulatory DNA to infer transcription factors (TFs) that may modulate transcription and chromatin state in different conditions. Most MEA methods determine motif enrichment independent of motif position within a sequence, even when those sequences harbor anchor points that motifs and their bound TFs may functionally interact with in a distance-dependent fashion, such as other TF binding motifs, transcription start sites (TSS), sequencing assay cleavage sites, or other biologically meaningful features. We developed motif enrichment positional profiling (MEPP), a novel MEA method that outputs a positional enrichment profile of a given TF’s binding motif relative to key anchor points (e.g. transcription start sites, or other motifs) within the analyzed sequences while accounting for lower-order nucleotide bias. Using transcription initiation and TF binding as test cases, we demonstrate MEPP’s utility in determining the sequence positions where motif presence correlates with measures of biological activity, inferring positional dependencies of binding site function. We demonstrate how MEPP can be applied to interpretation and hypothesis generation from experiments that quantify transcription initiation, chromatin structure, or TF binding measurements. MEPP is available for download from https://github.com/npdeloss/mepp.

## INTRODUCTION

Transcription factors (TFs) coordinate cellular transcriptional responses to external or changing signals (1). Motif enrichment analysis (MEA) allows researchers to infer the TFs responsible for altering gene expression or chromatin state in response to internal or external stimuli. MEA achieves this through quantifying the enrichment of TF binding motifs in regulatory element sequences that exhibit a measurable biological response of interest, such as chromatin opening, histone modification, TF binding, or transcription. Methods such as ATAC-seq, ChIP-seq, or csRNA-seq quantify these responses and are widely used to study transcription regulation (2–4).

Most MEA methods analyze biological sequences for the simple presence or absence of a motif without regard to the motif's position within the sequence. However, the position of TF binding motifs can play important biological roles (5). For example, some transcription factors play a role in directing the selection of TSS, or preventing ectopic TSS utilization (6). The position of TF binding motifs relative to other motifs can also be important for establishing functional regulatory modules and TF co-binding, as reflected in regulatory motif grammars (7–9).

Recent sequencing advances allow the definition of TSS and TF binding sites at base resolution, thus enabling analysis of the functional aspects of motif positioning. PRO-cap or csRNA-seq assays reveal and quantify nascent transcription start sites, providing high-resolution transcription initiation data in both genomic and temporal axes (4,10). As a recent method, csRNA-seq maps TSS by size selecting for short (20–60nt) RNA species, then enriching for RNA possessing a 7-methylguanosine cap on their 5′ end that is added immediately after initiation (11): The resulting RNAs represent freshly initiated transcripts that can be detected regardless of final transcript's stability, allowing csRNA-seq to generate profiles of TSS at both gene promoters and at transcribed distal regulatory elements (e.g. active enhancers) (4).

For analysis of DNA binding, recent methods such as ChIP-exo and ChIP-nexus pinpoint TF binding locations at high resolution (2,12). Other assays, such as ATAC-seq and MNase-seq, define cleavage sites in open chromatin or at nucleosome boundaries (3,13). Proper analysis of these high-resolution measurements of biological or functional features can provide a more precise characterization of nearby motif positions and their regulatory functions.

There is a need for methods that visualize and quantify the spatial relationships between TF binding motifs and biologically relevant anchor points such as TSS. CentriMo (14) and TFEA (15) provide bin- and quartile- based approaches to analyzing these relationships, but there remains an unfulfilled need for a method that uses score information directly. To fulfill this need, we have developed motif enrichment positional profiles (MEPP). MEPP identifies sequence motifs enriched at positions relative to biologically meaningful features, thereby integrating position as an additional layer of information. This provides the user with knowledge about which motif positions are optimal for context-specific binding site functions, in the form of a positional profile. Because this profile correlates relative positions of predicted binding sites with biological function, it can narrow down a search for the most functional binding sites from hundreds of base pairs to a local neighborhood: This refinement directly addresses the concerns of Wasserman and Sandlin's futility theorem, which states that almost all predicted binding sites lack function (16).

Input for MEPP comprises scored genomic sequences of uniform length. Scores for these sequences can come from a biological readout (e.g. transcription level measured by a sequencing assay). To contextualize the position of motifs, the sequences should be centered on a biologically meaningful position, for example the location of TSSs, cleavage sites, other sequence motifs, or other meaningful features. Rather than calculating a singular enrichment score for a motif, like standard MEA methods, MEPP calculates a position-dependent enrichment profile for each motif. In this profile, highly positive values at a position correspond to a stronger positive correlation of the motif presence with the biological score assigned to each sequence. By contrast, more negative values at a position correspond to a stronger negative correlation with the biological score. The resulting profile thus reveals positions of motifs that are most likely to activate or repress the scored biological features. In addition, MEPP visualizes the distribution of the motif across the input dataset as a 2D heatmap of the motif's strength and presence across both motif positions and sequence ranks (based on the assigned biological score). These results help identify not just relevant motifs, but the positional constraints of motifs that delineate context or position-dependent function. The score-based principle of this enrichment method further avoids issues with arbitrary threshold selection, while controlling for sequence bias.

## MATERIALS AND METHODS

### MEPP implementation

In order to visualize and quantify local enrichment of motifs, we developed and employed MEPP. The typical execution of MEPP occurs in five parts:

- Input data and pre-processing
- Motif heatmap generation
- Positional profile computation
- Per-motif visualization
- Motif dataset visualization

### Input data and pre-processing

MEPP accepts input comprising a series of uniform-length scored DNA sequences in the scored FASTA file format, where the sequence score follows after the sequence header, separated by a space. The score for each sequence resembles the score column of a bed-file, with its meaning dependent on the assay in question. For example, when analyzing csRNA-seq, the user may assign the score to TSS usage/csRNA-seq signal, or the log_2_ fold change in TSS usage between two experimental conditions (Supplementary Figure S1A). To simplify the generation of input data, we include a helper script, ‘mepp.get_scored_fasta’ which generates a scored FASTA file from a scored BED file and a reference genome FASTA file.

Degenerate sequences, sequences from repetitive regions, and sequences sampled from overlapping genomic intervals can negatively affect the interpretation of the MEPP results. We describe optional steps to filter out these sequences in the Supplemental Methods.

### Motif heatmap generation

Position weight matrices (PWMs) represent TF binding motifs as a matrix of nucleotide specificities. The match of a given DNA subsequence to a PWM occurs at variable strengths, quantified as the log-odds score of the match between subsequence and PWM (17–19). PWMs usually accompany a log-odds score threshold above which a subsequence is determined to be a match to the motif PWM (19).

**Figure 1.**
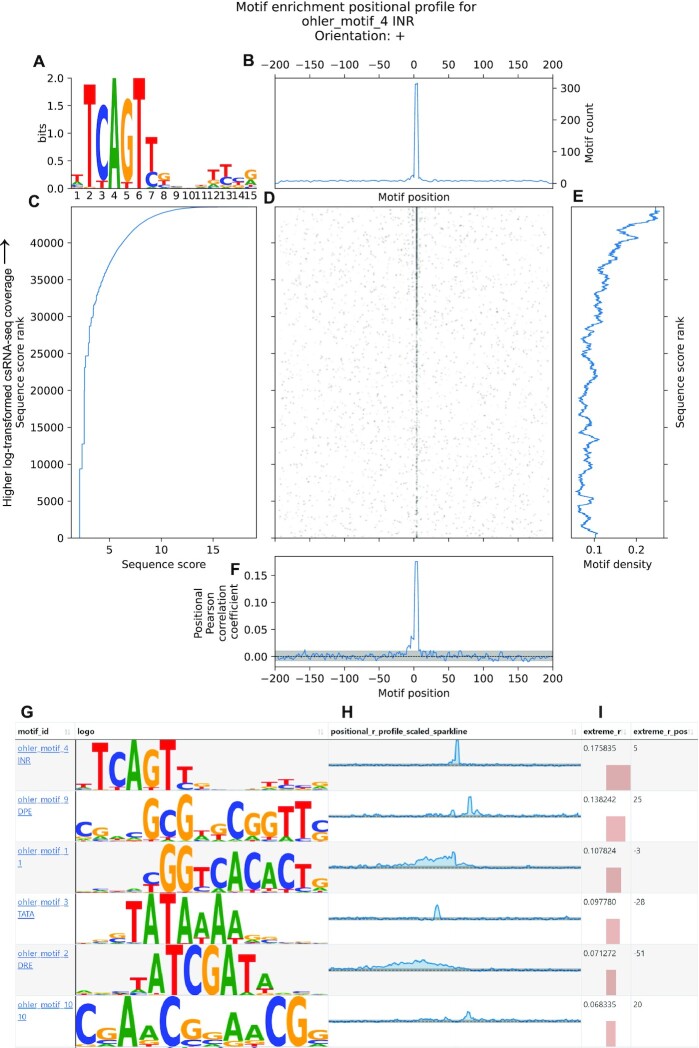
MEPP visualizes and quantifies core promoter motifs near Drosophila melanogaster transcription start sites. (**A**) Motif logo for the Drosophila Initiator motif. (**B**) Visualization of smoothed motif counts over each position across the 400 bp sequence, centered on the TSS quantified by csRNA-seq. (**C**) Line plot relating the sequence score (log-transformed csRNA-seq coverage) to the rank of the sequence score. Sequences are arranged in order of descending score in the dataset. (**D**) 2D motif heatmap summarizing motif occurrences across the whole dataset, with the horizontal axis indicating motif position, and the vertical axis indicating the rank of the sequence score. Each black spot represents a motif occurrence, with darker spots for stronger/more motifs in a downsampled neighborhood. (**E**) Line plot summarizing smoothed density of motifs across sequences in the dataset, with the vertical axis representing sequence score rank. (**F**) Visualization of the partial Pearson correlation values of motif strength/presence with score, quantified at each possible motif position surrounding the TSS, after controlling for sequence GC content. A 95% confidence interval is shaded in gray. (**G**) Partial screenshot of MEPP’s interactive table output. Motifs are identifiable in MEPP’s interactive table by motif ID and sequence logo. (**H**) Motif positional profiles are summarized using a sparkline visualization, allowing exploration of profiles at a glance. Pictured are the Initiator motif, TATA Box motif and DPE motif. (**I**) Extremes (minima, maxima) of motif positional profiles are summarized, including the values and where they occur relative to the sequence center.

MEPP accepts a list of motifs in JASPAR format (17). For each motif *j*, MEPP creates a convolutional model function *f_j_* that accepts a one-hot encoded DNA sequence *S_i_* and outputs log-odds match scores to the given motif (Supplementary Figure S1E). All sequences *S* are expected to have the same length. Using functions from the Motif Occurrence Detection Suite (MOODS) (19), we calculate a log-odds score threshold *b_j_* describing the minimum threshold log-likelihood match score for motif *j* under a given nucleotide background, pseudocount, and p-value threshold (Supplementary Figure S1E). Thus, given a motif *j* and sequence *i*, MEPP computes a heatmap row vector as:

$$\begin{equation*}{H_{i,j}}\; = {h_j}\left( {{S_i}} \right)\; = \;pad\left( {max\left( {0,\;{f_j}\left( {{S_i}} \right) - {b_j}} \right)} \right)\end{equation*}$$

where pad is a 0-value padding function that ensures *H_i,j_* and *S_i_* have the same length. When considering both orientations of a motif, MEPP will instead compute the row *H_i,j_* as:

$$\begin{equation*}{H_{i,j}}\; = \;max\left( {{h_j}\left( {{S_i}} \right),\;reverse\left( {{h_j}\left( {revcomp({S_i}} \right)} \right)} \right))\end{equation*}$$

where *reverse*(*X*) reverses an array of motif scores, and *revcomp*(*S_i_*) computes the reverse complement of one-hot sequence *S_i_*. When all sequences *S* are sorted according to score, the matrix of all rows *H_i,j_* over sequence indices *i* is the motif score heatmap *H_j_* for motif *j*. The central plot generated by MEPP displays the heatmap *H_j_* (Figure 1D) with the horizontal axis corresponding to motif position, and the vertical axis corresponding to each of the input sequences sorted in descending order based on their sequence scores. Motif position is measured from the center of sequences in *S* to the center of motif *j*. Rendering of the motif heatmap has additional considerations described in the Supplemental Methods.

To account for inexact motif positioning, we later optionally apply average pooling with a stride of 1 to the rows of the motif score heatmap *H_i,j_*. The size of the pooling window *w* can be modified to adjust the resolution of the positional profiles, and is computed as *w* = 1 + 2*m*, where *m* is a user-defined motif-margin. For high-resolution datasets that describe features at single nucleotide resolution, such as TSS found with csRNA-seq, the motif margin used should be small, usually 2 bp, but for lower-resolution datasets where the definition of the anchor may be less precise, such as the position of ChIP-seq peaks, a higher motif margin (e.g. 10 bp) may increase sensitivity. We apply 0-value padding to the data so that convolution and average pooling operations result in tensors matching the dimensions of the original one-hot encoded sequence. For simplicity, we refer to the smoothed form of row *H_i,j_* as *H_smoothed,i,j_*

### Positional profile computation

To calculate local motif enrichment at each sequence position across the dataset, at each position column *X* of the motif heatmap *H_smoothed,j_*, we calculate the partial Pearson correlation *p_XY·Z_* of the motif score matrix against the vector of sequence scores *Y*, while controlling for the vector of sequence-wide GC ratios *Z*. The resulting vector *P_XY·Z_* of positional correlation coefficients describes the enrichment of the motif across all positions. We term a motif's vector *P_XY·Z_* the positional profile or positional Pearson correlation for that motif. This enrichment method is comparable to that used by Analysis of Motif Enrichment (AME) (20), which by default calculates enrichment as the correlation between average motif match scores across each sequence and the sequence scores. For each motif, the positional profile *P_XY·Z_* is plotted across the same motif position axis as the central heatmap (Figure 1F).

In order to determine statistical significance, we use permutation testing to calculate positional profiles on multiple null permutations of the data. The permutation test shuffles sequence scores to break the relationship between motif position/presence and score. The resulting null enrichment profiles are used to derive confidence intervals and *P*-values for the scores in the positional profile. Confidence intervals are shaded in gray beneath the positional profile (Figure 1F).

We also calculate the count of motifs at each position summed up across the datasets. A rolling average with window size *w* smooths these values, which our method plots above the central motif heatmap of the MEPP visualization (Figure 1B).

### Per-motif visualization

For each motif, MEPP creates a plot with multiple subplots visualizing different aspects of the motif enrichment. These include the central heatmap (Figure 1D), positional profile (Figure 1F), and smoothed motif counts over positions (Figure 1B) previously described, as well as the motif logo generated by Logomaker (Figure 1A) (21). In addition, the left-hand-side plot displays the relationship between the rank of the sequence score vs. the score (Figure 1C), helping diagnose issues caused by non-normal score distributions that may throw off the correlation metrics. To contextualize the results a user might expect from non-positional score-based MEA, the right-hand-side plot displays the density of motifs as they occur along the dataset, smoothed along the sequence score rank axis for display (Figure 1E).

### Motif dataset visualization

MEPP also provides an interactive table for navigating to the profiles generated for each motif in a dataset (Figure 1G–I). For each motif, MEPP renders the positional profile and its confidence interval in a sparkline format (Figure 1H), alongside an illustration of the motif matrix itself (Figure 1G). The method identifies the extreme values of the positional profiles (Figure 1I) and records them alongside their confidence interval and associated *P*-values. To control for false positives, the Benjamini–Yekutieli (22) correction adjusts p-values by correcting across all positional *P*-values and all motifs; We use the correction implemented by statsmodels (23). MEPP renders the resulting table in HTML, augmented with interactive sorting and filtering features using the DataTables Javascript library (https://datatables.net/).

To aid in data exploration, MEPP renders a custom HTML output (Figure 3B–E), placing the motif matrices next to a heatmap and dendrogram displaying the positional profiles and their clustering hierarchy; This custom interactive clustermap displays motifs along the vertical axis. To keep output legible, we use interactive CSS to expand rows of the heatmap on mouseover.

MEPP clusters the motifs by their positional profiles using UPGMA hierarchical agglomerative clustering (https://doi.org/10.1007/978-1-4020-6754-9_17806) with a correlation clustering metric. The clustering assignments of each motif profile follow the defaults for scipy's ‘dendrogram’ function (Figure 3B) (24).

Both the table and clustermap HTML output generated by MEPP allow users to navigate to the individual MEPP plots for each motif through hyperlinks.

### Time complexity of MEPP

Because MEPP incorporates a visualization of the occurrences of *m* motifs over *n* sequences of length *l*, the creation of motif heatmaps must occur on the order of m*n*l. The clustering of motifs by their positional profiles similarly requires comparisons of profile similarity, on the order of *m*^2^*l. Thus, the overall time complexity occurs on the order of O(m*n*l+*m*^2^*l), but in practice, this is dominated by the first term O(m*n*l), which is linear for the size *n* of appreciably large datasets of scored sequences.

### Public data used and analyzed

We used MEPP to analyze multiple public datasets. For each dataset, we sample sequences surrounding measured events from the relevant sequencing assay, and score these sequences according to either normalized read count coverage or the log_2_ fold change when comparing conditions. We then input the resulting scored sequences to MEPP. For convenience, these analyses are summarized in Table 1, while full analysis details are provided in the Supplemental Methods. Access information and lab attribution for public data used from other studies is recorded in Supplemental Table S6.

**Table 1.** Public data used in this study

**Discussion section title**	**Methods/ supplemental section title**	**Sequencing assay**	**Measured event**	**Sequence center**	**Sequence length (bp)**	**Comparison**	**Sequence scores**	**Score polarity (meaning of higher scores)**	**Genome**	**Dataset accession(s)**
MEPP visualizes and quantifies positions of core promoter motifs	Analysis of *Drosophila melanogaster* TSS	csRNA-seq	Nascent transcription initiation	Nascent transcription start sites	400	Nascent transcription vs. none	log-transformed fragment 5' end coverage	Higher transcription	dm6	GSE203135
MEPP visualizes ChIP-seq peaks	MEPP analysis of GATA1 ChIP-seq	ChIP-seq	GATA1 binding	MACS2-called peak summits	400	ChIP vs. input	log_2_ Fold Change GATA1 ChIP-seq vs input	Greater GATA1 ChIP-seq signal	hg38	ENCSR000EFT (GATA1 ChIP-seq), ENCSR000EHM (Control)
MEPP visualizes cell-type specific TF binding motif spacing	Analysis of differential chromatin accessibility between cell types	ATAC-seq	Cleavage of accessible chromatin	GATA1 binding motifs	200	HCT116 vs. K562	log_2_ Fold Change HCT116 versus K562 ATAC-seq	Greater accessibility HCT116	hg38	ENCSR483RKN (K562 ATAC-seq), ENCSR872WG (HCT116 ATAC-seq)
MEPP identifies helical spacing for motifs associated with cooperative Nanog binding	Analysis of Nanog motif binding in *Mus musculus*	ChIP-seq	Nanog binding	Nanog binding motif	400	Normalized coverage	RPKM-transformed Nanog ChIP-seq coverage	Higher coverage	mm10	GSE144577
MEPP visualizes differing positional specificities of TF binding assays	MEPP Analysis of Mouse ChIP-nexus and ChIP-seq	ChIP-seq	Nanog binding	MACS2-called peak summits	400	Binding signal vs. background	MACS2 peak signal	Higher ChIP-seq signal	mm10	GSE137193
		ChIP-nexus	Nanog binding				MACS2 peak signal	Higher ChIP-nexus signal		
		ChIP-nexus	Nanog binding	5' read end locations		Normalized coverage	rlog-normalized fragment 5' end coverage	Higher ChIP-nexus 5' end coverage		
MEPP yields concordant profiles for assays of differential LPS response	Differential csRNA-seq analysis	csRNA-seq	Nascent transcription initiation	Nascent transcription start sites	400	KLA-stimulated vs. control	log_2_ Fold Change KLA versus control nascent transcription	Higher transcription in KLA stimulation	mm10	GSE135498
	Differential cleavage site analysis	ATAC-seq	Cleavage of accessible chromatin	Cleavage sites		LPS-stimulated versus control	log_2_ Fold Change LPS versus control nascent cleavage	Higher transcription in LPS stimulation		GSE119693
		MNase-seq								

## RESULTS

### MEPP visualizes and quantifies positions of core promoter motifs

To demonstrate the utility of our method in identifying known positional dependencies for DNA motifs, we analyzed transcription start sites (TSSs) in *Drosophila melanogaster* embryo cells. Using capped small (cs)RNA-seq, we identified 44 877 high confidence TSSs (read count >3 while controlling for background input, repetitive DNA content, and overlapping sites, see Supplemental Methods). We extracted sequences covering ±200 bp from each TSS and scored them by the log-transformed csRNA-seq coverage of their TSS centers, where higher scores correspond to TSS with higher rates of initiation. We then ran MEPP using a library of motifs previously found to be enriched in *Drosophila* promoters (25), focusing on the positioning of the TATA-box, Initiator (Inr), and Downstream Promoter Element (DPE) motifs, which are expected to appear upstream, on, and downstream of TSS, respectively.

MEPP visualizes a motif's occurrences in a scored sequence dataset in one figure comprising multiple plots with aligned axes (Figure 1). The central 2D motif heatmap allows more direct visualization and qualitative evaluation of motif distributions across the dataset: the presence of the Inr motif on the TSS, in the sequence center, is clearly visible and is more well defined for TSS with greater transcriptional activity (Figure 1D). The right-hand plot of motif density over sequence ranks also reflects the association of pronounced Inr motifs with greater TSS activity, and resembles the data as it would appear to a motif enrichment method using the zero-or-one-occurrence per sequence (ZOOPs) model (Figure 1E). However, it is the enrichment positional profile plotted at the bottom that summarizes the positionality of this enrichment (Figure 1F): This plot illustrates that the association of Inr motif strength/presence with TSS strength is most positively correlated at the sequence center, on the TSS itself, which matches expectations. Similarly, the TATA-box motif profile peaks upstream of the TSS at −28 bp, while the DPE motif profile peaks 25 bp downstream of the TSS (Supplementary Figure S2A, B). Positions are reported using the distance from the center of the motif to the center of the sequence (which is defined here to be the TSS).

MEPP performs an analysis of multiple motifs for a dataset and summarizes them in an interactive table (Figure 1G–I). The top 2 results for the most extreme positional profile values corresponded to profiles for the Inr and DPR motifs, while 4th result corresponded to the profile for the TATA-box. The results table also describes the location of these extreme values across sequences centered on the TSS (as determined by the position of the largest absolute values in the profile). As expected, maximum correlation of the TATA-box motif with transcriptional initiation occurs upstream at -28 bp relative to the TSS, while the DPE’s maximum correlation occurs downstream at +25 bp relative to the TSS. This is consistent with the known positioning characteristics of these core promoter elements (26,27). Thus, we demonstrate how MEPP’s multiple readouts recapitulate ground truths about promoter organization from a high-resolution nascent transcriptional assay.

### MEPP visualizes ChIP-seq peaks

**Figure 2.**
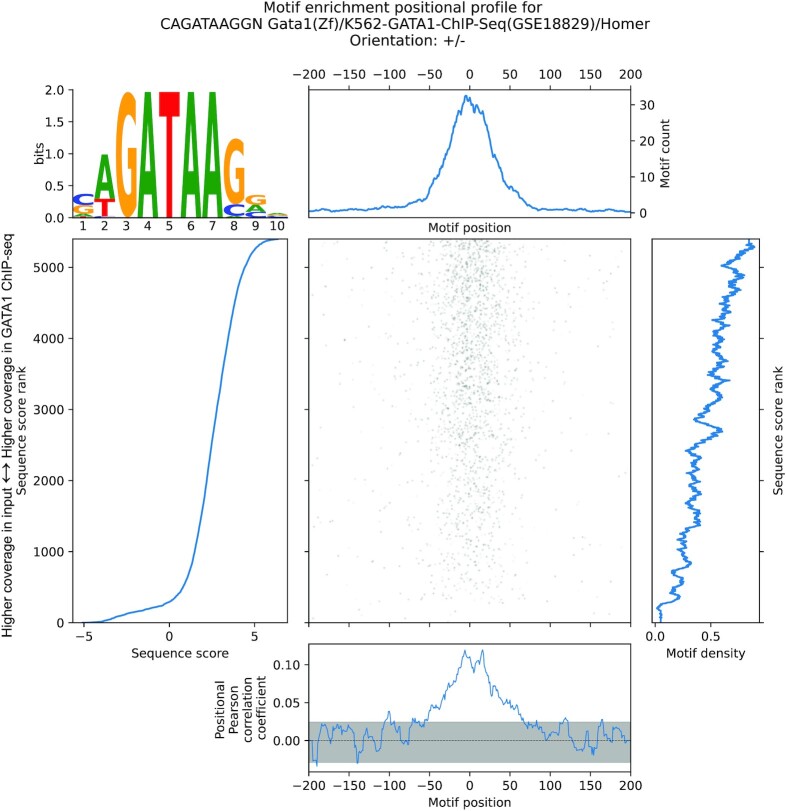
MEPP visualizes and quantifies the GATA1 binding motif in GATA1 ChIP-seq binding sites. MEPP plot for the GATA1 motif, on sequences ±200bp of GATA1 ChIP-seq peak centers sampled from the hg38 reference genome, which are scored by differential ChIP-seq coverage (log_2_ fold change of GATA1 ChIP-seq versus input control).

To demonstrate our method's ability to visualize known motif content in more general sequencing assays with less exact positioning, we analyzed ChIP-seq peak summits for GATA1 in K562 cells. We used MACS2 on ENCODE GATA1 ChIP-seq alignment files and the corresponding Control ChIP-seq alignment files to extract over 5K non-overlapping sequences sampled ±200 bp from GATA1 ChIP or Control ChIP summits and scored by the log_2_ fold change between GATA1 ChIP and control. MEPP analysis on these scored sequences found centrally positioned enrichment of the GATA1 motif correlated with higher coverage in GATA1 ChIP over control, as expected (Figure 2).

Unlike the previous analysis, this enrichment profile reflects positional sequence matches regardless of the orientation of the GATA1 motif. The maximum correlation signal in the positional profile provided by MEPP is less sharp compared to the analysis of core promoter elements relative to single nucleotide-resolution TSS, reflecting the less positionally specific nature of the ChIP-seq assay compared to the csRNA-seq assay. Similar to the previous result on TSS, this demonstrates that MEPP can identify known motif distribution patterns correctly, even when the assay in question has less distinct positional landmarks. This marks its utility in characterizing such assays as a visualization and quality control tool.

### MEPP visualizes cell-type specific TF binding motif spacing

To demonstrate MEPP’s ability to identify cell-type specific regulatory grammars, we analyzed the occurrence of motifs surrounding GATA1 binding motif sequences in K562 and HCT116 cells. Instead of using features of NGS profiling to determine analysis anchors (e.g. TSS, ChIP-seq peak summits), here we anchor our analysis on GATA motifs and analyze how the presence of other nearby TF motifs are associated with regulatory element activity. Over 500K GATA1 binding motifs appear in the human genome, but these are not in equally accessible chromatin, especially across cell types. To determine if increased cell-type specific chromatin accessibility associates with a spacing preference between GATA1 and other motifs, we used MEPP to analyze the positions of other binding motifs surrounding GATA1 binding motifs. We extracted genomic sequence ±100 bp around GATA1 binding motifs, then scored these sequences by the log_2_ fold change of chromatin accessibility between HCT116 and K562 cell types; High scores corresponded to higher accessibility in HCT116 than in K562, as measured using ATAC-seq. These scored sequences comprised our input to MEPP for this analysis.

**Figure 3.**
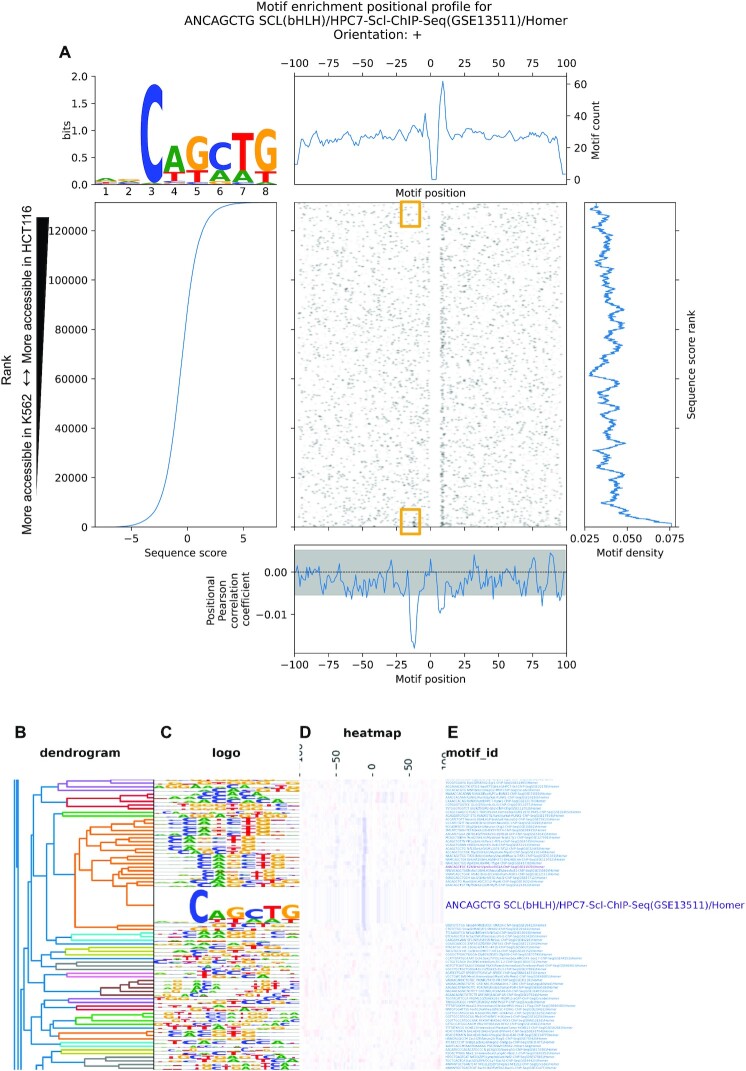
MEPP visualizes and quantifies the SCL/TAL1 binding motif near GATA1 binding motif locations. (**A**) MEPP plot for the SCL/TAL1 binding motif, on sequences ±100 bp of GATA1 ChIP-seq peak centers sampled from the hg38 reference genome, which are scored by differential chromatin accessibility score (log_2_ fold change of HCT116 over K562, by ATAC-seq). Yellow boxes indicate extrema of the heatmap where SCL motif presence contributes to the enrichment profile's minima. (**B**) Dendrogram illustrating cluster membership of motifs characterized by enrichment positional profiles. (**C**) Motif logos represented in compacted form alongside enrichment profiles, with full logos visible on mouseover. (**D**) Heatmap visualizing motif enrichment profiles as rows of color bars, with red, white, and blue coloration signifying positive, zero, and negative correlation with sequence score. (**E**) Motif names with hyperlinks to full MEPP plots, with enlarged font scaling on mouseover for readability

The transcription factor GATA1 plays a key role in hematopoiesis and erythroid gene expression (28). After GATA transcription factor motifs, the top results for significant positional profiles in MEPP featured binding motifs for bHLH transcription factors, exemplified by SCL. The heatmap for the SCL motif (also known as TAL1) indicates preferential positioning of this motif around 12 bp upstream of the GATA1 motif, as measured from the center of SCL motif to the 5′ end of the GATA motif (Figure 3A). This is consistent with the approximate requirements for binding of a complex assembled by Lmo2 including SCL and GATA1 (28), and is consistent with previous reports characterizing composite GATA:Ebox motifs bound by the factors during erythroid maturation (29). The enrichment profile generated by MEPP indicates that this positioning of the SCL motif has enrichment surrounding GATA motif sites with greater chromatin accessibility in K562 cells, but not HCT116 cells (Figure 3A), as indicated by the negatively scored valley in the profile at that upstream position. This is consistent with the erythroleukemia origin of K562 cells, where GATA1 and SCL/TAL1 transcription factors play important roles in hematopoietic differentiation of the erythroid lineage. Although the motif heatmap indicates that this profile derives from motifs in a relatively small section of the heatmap (Figure 3A, yellow squares), these sections still reflect motifs present in thousands of the most extremely scored sequences.

MEPP visualizes and clusters the profiles generated for multiple motifs as an interactive HTML clustermap (Figure 3B–E). This allows users to determine regimes of positional dependencies shared by similar motifs. For example, the SCL motif clusters with similar basic helix-loop-helix binding motifs. Unlike conventional non-interactive heatmaps, our approach to visualization allows users to expand motif logos and text, as well as to click through hyperlinks to the full MEPP profile. This allows for determination of profile similarities across a full motif set at a glance, rendering no singular row permanently unreadable. This combination of novel interactive visualization techniques and positional, score-based motif enrichment is unique to MEPP’s approach, and enables users to identify cell-type specific regulatory grammar.

In order to evaluate MEPP’s ability to identify positionally relevant motifs compared to other approaches, we analyzed the same scored sequences using TFEA (15), and the upper and lower 10% of the scored sequences using CentriMo (14) and HOMER (30). CentriMo and TFEA incorporate the analysis of motif positions in their results, while HOMER serves as a general motif analysis tool to compare against. Both CentriMo and TFEA produced results that confirm SCL and other bHLH motifs are positionally enriched near K562 accessible GATA1 motifs (Supplementary Figures S3 and S4). However, for the purposes of discovery, only MEPP and CentriMo reported SCL or other bHLH motifs among the top motifs in its output result tables (Table S5). For example, TFEA and HOMER identified motifs bound by ETS family TFs ahead of bHLH motifs in their results. ETS motifs are generally enriched in the vicinity of the GATA1 motifs in K562 open chromatin, but lack specific spacing relationships. These differences reflect the strategies each method uses to identify biologically interesting motifs, which often rely on position-independent enrichment (e.g. HOMER). This distinction is important since results reported by a motif enrichment method that do not appear at the top of the results table are often ignored, impacting downstream interpretation.

In order to evaluate methods of describing positional enrichment, we focus on positional profiles generated by MEPP and CentriMo (14). Because CentriMo takes contrasting sets of sequences as input, rather than continuously scored sequences, we submitted the lower 10% of scored sequences as the ‘positive’ set for enrichment, and the upper 10% as a contrasting ‘negative’ set. The resulting local enrichment plot (Supplementary Figure S3) yields a profile that does not differentiate between a motif being simply prevalent at a position, or more enriched in the ‘negative’ set of sequences. Instead, this profile has two positive peaks, consistent with the peaks in MEPP’s plot of motif counts over positions across sequences (Supplementary Figure S3). While a second profile is plotted as a dashed line reflecting enrichment in the negative set of sequences, its interpretation relies on the selection of the negative set of sequences, and a quantitative summary requires downstream comparison against the profile for enrichment in the positive set (Supplementary Figure S3). This underscores a key difference in MEPP’s enrichment profile output from current methods like CentriMo: Rather than only quantifying a motif's positional prevalence in a thresholded selection of a dataset, MEPP, quantifies motif's positional relevance towards a higher or lower scoring sequence, as measured by the local correlation of motif score and sequence score. In addition, CentriMo only accounts for the position of the best match to the motif within a sequence, while MEPP quantifies and visualizes all motif instances within a sequence. Prioritizing only the strongest match to a binding motif can be counterproductive to identifying tissue-specific motif grammars, which can compensate for weaker binding motifs (9).

### MEPP identifies helical spacing for motifs associated with cooperative Nanog binding

To demonstrate MEPP’s ability to identify complex relationships between motifs that have roles in cooperative TF binding, we performed an analysis of Nanog binding in mouse embryonic stem cells (ESC). The Nanog motif is relatively common in the genome, but not all instances of this motif are bound. The Nanog motif instances that are bound often have varying rates of association with Nanog as measured by ChIP-seq. To identify other motifs near Nanog motifs that have positional specificities in their ability to influence Nanog binding, we performed a MEPP analysis of Nanog motif sites across the mm10 reference genome. We scored Nanog motif sites by their Nanog binding activity as quantified by Nanog ChIP-seq in mouse embryonic stem cells (mESCs) (31). The analysis processed over 3M sequences sampled ±200 bp of Nanog motif sites, after overlapping interval deduplication and filtering out sequences containing 50% repetitive or degenerate bases as annotated byRepeatMasker.

**Figure 4.**
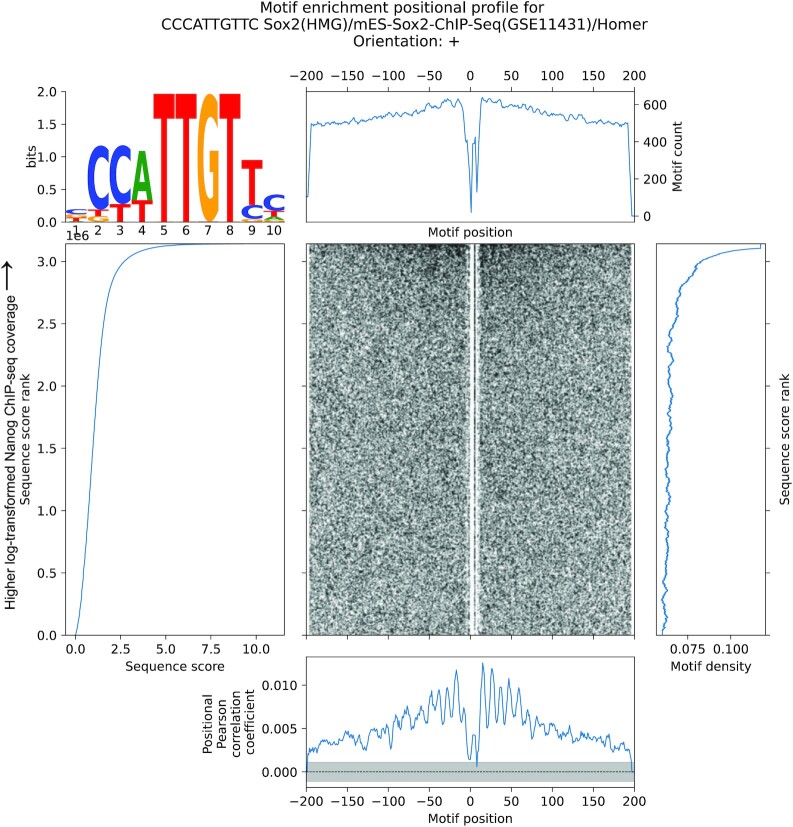
MEPP visualizes and quantifies the Sox2 motif near bound Nanog motif sites. MEPP plot for the Sox2 motif, on sequences ±200 bp of GATA1 ChIP-seq peak centers sampled from the mm10 reference genome, which are scored by log-transformed Nanog ChIP-seq coverage.

MEPP analysis showed that motifs bound by pluripotent transcription factors often revealed helical spacing preferences to Nanog motifs bound by Nanog in mESCs. The MEPP plot for enrichment of Sox2 motifs surrounding central Nanog motifs reveals periodicity in the enrichment positional profile with a period of ∼10 bp (Figure 4). This periodicity is less visible when simply plotting Sox2 motif counts over positions relative to Nanog (Figure 4). Positive peaks in the enrichment positional profile represent a stronger local correlation of Sox2 motif strength/presence with Nanog binding at those periodically spaced positions, suggesting that cooperative binding of Sox2 and Nanog depends on a helical syntax that preserves the relative rotational positions of the factors along the DNA. Other approaches leveraging machine learning models have also found helical binding periodicities between Nanog and Sox2 motifs (32,33). However, our method does not require the training or interpretation of machine learning models, but yields concordant results. Importantly, due to the overlapping interval deduplication step in the data preprocessing, our results do not reflect repetition of the Nanog motif around itself, ensuring that these findings are not due to e.g. a single Sox2 motif appearing near multiple Nanog motifs that are spaced periodically with each other, as might occur in unannotated repetitive genome sequence. By combining MEPP with careful preprocessing, we demonstrate the ability to identify properties of motif spacing more complex than single peaks of positional enrichment.

### MEPP visualizes differing positional specificities of TF binding assays

To demonstrate the effect of assay type positionality on the positional profiles derived by MEPP, we analyzed ChIP-nexus and ChIP-seq Nanog binding assays in mouse embryonic stem cells, as carried out by Avsec *et al.* (32). ChIP-nexus assays use exonucleases to precisely map the locations where crosslinked proteins protect the DNA, suggesting that ChIP-nexus peaks should provide greater precision than ChIP-seq peaks with respect to binding motifs (2). MEPP analyzed 39K Nanog ChIP-nexus peaks and over 28K Nanog ChIP-seq peaks, using sequence sampled from ±200 bp of each peak summit and scores taken from the signal values in the MACS2 narrowpeak calls. To account for the lack of strand specificity in ChIP-seq, MEPP correlated sequence scores against both forward and reverse orientations of each motif.

**Figure 5.**
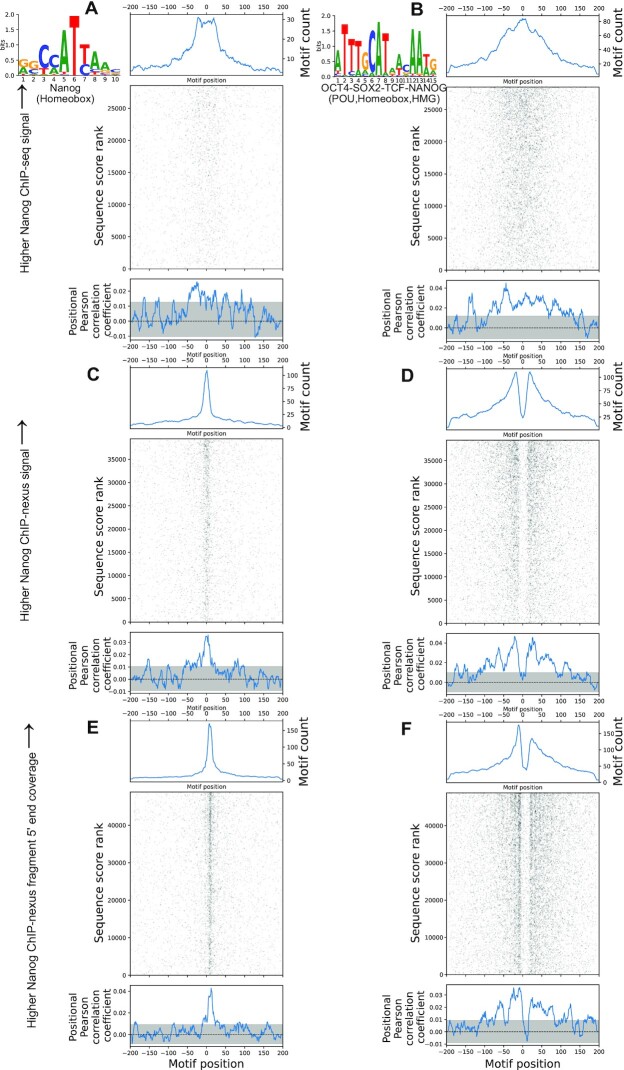
MEPP differences in positional specificity between ChIP-seq and ChIP-nexus (**A**) MEPP plot for Nanog binding motif, on sequences ±200 bp of Nanog ChIP-seq peak summits sampled from the mm10 reference genome, scored by MACS2 signal value for each peak. (**B**) MEPP plot for Oct4–Sox2–TCF–Nanog composite binding motif, on sequences ±200 bp of Nanog ChIP-seq peak summits sampled from the mm10 reference genome, scored by MACS2 signal value for each peak. (**C**) Same, as (A), but for sequences sampled and scored from Nanog ChIP-nexus peak summits. (**D**) Same, as (B), but for sequences sampled and scored from Nanog ChIP-nexus peak summits. (**E**) Same, as (A), but for sequences sampled and scored from Nanog ChIP-nexus fragment 5′ ends found and scored using an alternate HOMER analysis pipeline adapted from use on csRNA-seq. (**F**) Same, as (B), but for sequences sampled and scored from Nanog ChIP-nexus fragment 5′ ends found and scored using an alternate HOMER analysis pipeline adapted from use on csRNA-seq.

As expected, the Nanog motif positional profiles derived from Nanog ChIP-nexus peaks showed greater positional specificity, with the positional profile indicating a positive peak centered directly on the peak summit (Figure 5C). In contrast, the Nanog motif positional profile from the Nanog ChIP-seq experiment indicates a broader, less well-defined central peak that does not rise as far above the 95% confidence interval (Figure 5A). Additionally, positional profiles for the Oct4–Sox2–TCF-Nanog composite motif follow a similar pattern, with the Nanog ChIP-nexus derived profile having enough granularity to resolve two peaks on either side of the peak summit, as opposed to the broader profile reflected from the Nanog ChIP-seq experiment (Figure 5B,D). The motif heatmap visualization offered by MEPP enables researchers to visualize the underlying two-dimensional distribution of motifs surrounding each experiment's peak summits, providing further feedback on the positional properties of each dataset. Thus, MEPP results capably reflect the positional specificities of different sequencing assays, allowing both quantitative and qualitative feedback on sequence features enriched in the surrounding assay peak summits.

The positional specificity visualized by MEPP is further enhanced using analysis methods that leverage the positional information provided by the ends of the reads, such as those developed for csRNA-seq. To demonstrate, we re-analyzed the ChIP-nexus data that follows the example of csRNA-seq, by identifying and scoring prominent ChIP-nexus 5′ protection boundaries from the 5′ ends of the Nanog ChIP-nexus reads. This alternative analysis identified 48K potential binding sites, scored by the (DESeq2) rlog-transformed coverage of the Nanog ChIP-nexus 5′ read ends (34). MEPP was used to analyze sequence ±200 bp of these binding sites for motif enrichment. The resulting profiles for the Nanog motif and the Oct4–Sox2–TCF–Nanog are similar to the previous Nanog ChIP-nexus experiment (Figure 5E, F). However, the enhanced specificity and data density appears as visible vertical striations of motif presence on the central motif heatmaps, which provides a clearer profile peak center in the case of the Nanog motif profile (Figure 5E). Thus, MEPP results reflect specificities from both assay types and analysis approaches, providing both quantitative and qualitative feedback to researchers developing or refining methods for assay or analysis.

### MEPP yields concordant profiles for assays of differential LPS response

**Figure 6.**
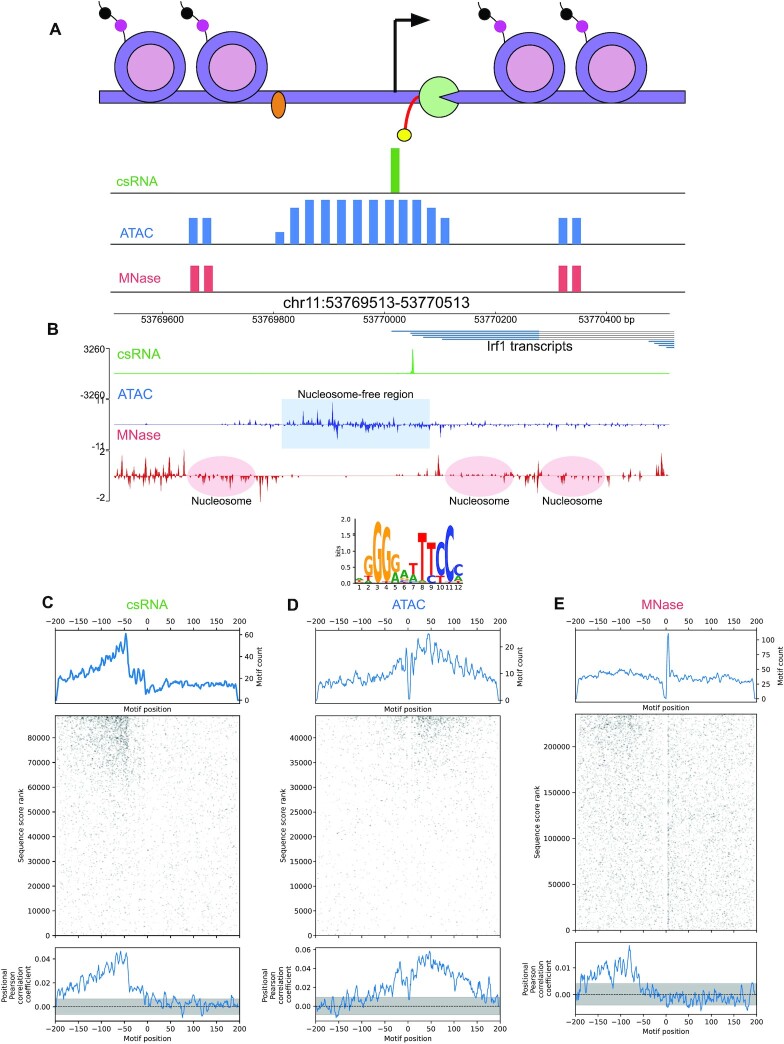
MEPP Plots summarize NF-κB motif enrichment across csRNA-/ATAC-/MNase-seq TSS/Cleavage sites. (**A**) Diagram illustrating read coverage from csRNA-seq (Green), ATAC-seq (Blue), and MNase-seq + H3K27ac ChIP (Red) experiments. Adapted from Tsompana and Buck (36). csRNA-seq assays nascent TSS from 5′ capped short RNA transcripts, while ATAC-seq and MNase-seq assay open chromatin. MNase-seq from Comoglio et al. includes immunoprecipitation of H3K27ac. (**B**) Integrated Genome Browser visualization of coverage from 5′ ends of csRNA-seq, ATAC-seq, and MNase-seq reads near the *Irf1* transcription start site in mm10. (**C**) MEPP plot for NF-κB binding motif, on sequences centered on csRNA-seq derived TSS, and scored by differential TSS nascent transcription between 1 h LPS stimulation versus 0 h control. (**D**) Similar as (**B**), but for sequences centered on ATAC-seq cleavage sites, and scored by differential 5′ read coverage between 1 h LPS stimulation versus 0 h control. (**E**) Similar as (C), but for sequences centered on H3K27ac MNase-seq cleavage sites.

To demonstrate the applicability of MEPP to multiple types of sequencing experiments, we performed a differential analysis of TSS measured by csRNA-seq and cleavage sites from ATAC-seq and MNase-seq experiments. These experiments compared the state of mouse bone marrow-derived macrophages (BMDMs) before and after 1 h of LPS stimulation (4,35). which activates innate immunity pathways by triggering Toll-like receptor 4 (TLR4) signaling. In each experiment, sequences were sampled from ±200 bp of genomic coordinates taken from the 5′ ends of reads: in csRNA-seq, these represent TSS, while in ATAC-seq and MNase-seq, these represent cleavage sites for accessible DNA by the assay's respective enzyme (Figure 6A, B, adapted from Tsompana & Buck 2014) (3,13,36). In the case of MNase-seq, digested chromatin was further ChIPed for H3K27ac, reflecting transcriptionally active nucleosomes (35). All TSS/cleavage sites and their associated sequences were scored by log2 fold change comparing pre- and post-stimulation coverage as calculated by DESeq2.

The transcription factor NF-kappa B (NF-κB) is known to induce strong changes in transcription in response to activation of TLR4 by LPS (37). Thus, MEPPs for the NF-κB binding motif all feature concordantly positive peaks. In the csRNA-seq derived MEPP analysis of this motif, there is a clear positional peak 58bp upstream of TSS implying NF-κB binding to this position potently initiates transcription after activation (Figure 6C). Similarly, in the H3K27ac MNase-seq analysis, the MEPP for the NF-κB binding motif exhibits a positive peak at 81 bp upstream of the MNase cleavage site (Figure 6E), indicating NF-κB binding likely increases histone acetylation on nucleosomes or repositions acetylated nucleosomes with their edge ∼80 bp from of the NF-κB motif. Notably, this peak is distinct from the location where the same motif is most prevalent in sequence, just downstream of the cleavage site. Such a distinction underscores the ability of MEPP to distinguish motif relevance to biological signal, as opposed to motif prevalence across a set of sequences agnostic to biological signal.

Unlike the profiles for TSS and nucleosome edges, ATAC-seq derived MEPP analysis of the NF-κB binding motif revealed a strong preference approximately 45 bp downstream of the Tn5 cleavage site, generally placing NF-κB-DNA contacts on the fragments isolated in the ATAC-seq assay. There is also a positive association of NF-κB binding just upstream of the cleavage site, suggesting NF-κB binding may enhance the accessibility of sizable regions surrounding the NF-κB motif. (Figure 6A, B, D) (38). Similarly, there is positive motif enrichment both up and downstream of the central cleavage site, reflecting ATAC-seq read coverage surrounding a TF binding footprint. However, these profiles are still concordant with increased enrichment of the NF-κB motif in regulatory regions more accessible after LPS stimulation and its role in innate immune response. Thus, while peaks in the NF-κB motif profiles have concordant characteristics, differences in the profiles still reflect meaningful distinctions between the reads selected and sequenced for each assay. Such distinctions would not appear in analyses that report enrichment scores for motifs that do not take motif position into account, highlighting an advantage of MEPP’s positional approach to motif enrichment.

## DISCUSSION

MEPP correlates the log-odds scores of a motif with biologically relevant measurements as a function of the motif's position to identify spatial relationships in regulatory DNA. In contrast, many MEA methods such as MEIRLOP and HOMER treat motif presence within a sequence under a zero-or-one-occurrence-per-sequence (ZOOPs) model: For enrichment, a motif is either present or absent (30,39). This ignores how a motif may occur at multiple positions within a sequence and leads to methods that cannot describe positional dependencies of binding site function. Such positional dependencies may hold relevance when an experiment samples sequences from the genome surrounding biologically significant features, such as transcription start sites. By correlating motif presence at multiple positions in the sequences surrounding relevant features, MEPP enables positional profiling of motif enrichment alongside a structured visualization system that illustrates motif prevalence in the dataset. These results recapitulate known relationships such as the positioning of core promoter elements surrounding drosophila melanogaster TSS, and are capable of revealing more complex relationships including periodicities in motif positioning where a scatterplot/heatmap visualization does not provide enough clarity.

When applied to sequences surrounding GATA1 motifs, we find that our method recovers the positional relevance of SCL-to-GATA1 motif spacing to K562 cells, a result supported by the previous characterization of ternary complex formation on a composite GATA:E-box motif (29). We demonstrate the ability of MEPP to summarize the positional enrichment of all motifs in a dataset, and present them in a novel interactive clustermap format. The clustermap allows the identification of locally co-enriched motifs, such as those with similar consensus sequences, or those that may comprise sub-motifs for binding a larger cis-regulatory mechanism, such as the GATA-SCL motifs for an Lmo2-bridged binding complex. Thus, MEPP’s ability to visualize correlated positional relevance of motifs at a glance allows researchers to quickly observe transcriptional regulation mechanisms beyond single motifs, and to better contextualize results for single motifs.

When applied to multiple sequencing assays that present biologically relevant positioning features, such as csRNA-seq, ATAC-seq, or MNAse+ChIP-seq, we find that MEPP yields concordant profiles whose differences reflect the biochemical specificities of the assays analyzed. Each of these assays produce reads describing biological phenomena such as nucleosome edges or transcription initiation at single nucleotide resolution that MEPP can leverage to investigate the roles that transcription factors play in regulating these phenomena. This can prove invaluable when describing multiple functions of regulatory sequences.

We find that unlike most motif analysis software, which can plot the prevalence of a motif in a dataset of sequences, MEPP plots the positional relevance of motifs along a continuous score. The use of signed enrichment coefficients with a signed score allows researchers to investigate regulatory region sequences that vary between two extremes quantifiable by an assay-based score, such as those exhibiting cell-type- or stimulation-specific expression. While users could run similar analyses by analyzing quantiles or otherwise stratified bins of regulatory region sequences, these still require the user to select thresholds to partition the sequences according to best practices, which are not guaranteed when analyzing novel measures of biological activity. MEPP’s motif heatmaps can assist in this task, allowing researchers to visualize motif presence along two dimensions of position and assay-based score, while avoiding overplotting effects.

This transparency mitigates the risk of being misled by non-specific local motif prevalence. Similarly, MEPP plots the relationship between assay scores and sequence ranks, avoiding the risk of selecting non-informative thresholds for a score distribution. Thus, when taken together, all elements of a MEPP plot remain powerful in informing decisions for subsequent analyses.

We have demonstrated MEPP as a novel means of quantifying and visualizing the positional relevance of a motif across multiple centered genomic sequences. Similar to our previous work with MEIRLOP, MEPP is usable by scoring genomic regions across a continuum of scores reflecting two extremes of biological interest. Unlike other methods of performing positional motif enrichment, MEPP identifies local motif enrichments towards either extreme, with the sign reflecting a motif's association with higher or lower scores. MEPP currently functions with a fixed motif library. However, the underlying convolutional network architecture lends itself easily to future work for recognizing and assembling de novo motifs based on correlated positional profiles.

## DATA AVAILABILITY

All raw data generated for this study can be accessed at NCBI Gene Expression Omnibus (GEO; https://www.ncbi.nlm.nih.gov/geo/) accession number GSE203135. This work uses human cell line data from the ENCODE Project (40,41).

The code for MEPP is available from its Github repository at https://github.com/npdeloss/mepp, and can be installed through pip, via the command line: pip install git+https://github.com/npdeloss/mepp@main.

## Supplementary Material

lqac075_Supplemental_Files
