# Supplementary material for: MEPP: more transparent motif enrichment by profiling positional correlations
Source: NAR Genom Bioinform. 2022 Oct 17;4(4):lqac075. doi: 10.1093/nargab/lqac075 (PMC9575187; doi:10.1093/nargab/lqac075)
Supplement: lqac075_Supplemental_Files [file lqac075_supplemental_files.zip › MEPP Manuscript Revision.supplement.docx]

### **Supplementary Methods**

#### **Filtering of degenerate and repetitive sequences**

To filter out degenerate sequences, we remove sequences which possess more than a user-selectable threshold percentage of degenerate sequence content (not strictly A, C, G, or T). The resulting thresholded sequences are then one-hot encoded for use with convolutional models later in the analysis.

When used with soft-masked genomes that use lowercase bases to annotate repeats, MEPP considers these lowercase bases equivalent to the degenerate nucleotide code “N”. Users may bypass this behavior by processing the input file with awk’s toupper function.

#### **Overlapping Interval deduplication to filter genomically overlapping sequence**

If sequence from multiple overlapping genomic intervals is input into MEPP, this can lead to artificially inflated motif periodicities, as the same genomic subsequence that matches a motif can be repeated multiple times at shifted positions in the dataset. An example of this occurs when analyzing sequence sampled from +/- 200bp of TSS, when multiple TSS appear clustered less than 200bp from each other. To remedy this, we use bedtools cluster [(1)](https://paperpile.com/c/mx3Imw/u8Lp) to label groups of overlapping genomic intervals, then only select the most informative interval from the group; this is typically the interval with the most coverage from the assay, e.g. the strongest of multiple neighboring TSS. We then extract sequences and scores for this “overlapping interval deduplicated” set of intervals, and input these scored sequences into MEPP. Overlapping interval deduplication may be undesirable when studying effects that rely on a feature’s overlapping nature, e.g. spacing of a motif with neighboring instances of itself. In these scenarios, users must avoid over-interpretation of artificial periodicities stemming from overlapping features.

#### **Motif heatmap downsampling**

Naive downsampling of the motif heatmap to compress the vertical axis would render motif instances invisible: Pixels containing positive matches for a motif would become averaged with surrounding pixels containing no matches for a motif. To counter this, MEPP uses a local maximum function to downsample the original dimensions of the motif heatmap (N sequences x M positions) to MEPP’s display resolution.

#### **Analysis of Drosophila melanogaster TSS**

To identify TF binding motifs with position-dependent functions associated with transcription initiation, we performed csRNA-seq to map initiating transcripts in Drosophila melanogaster embryos. We started with cell culturing and csRNA-seq for data acquisition. This was followed by a csRNA-seq bioinformatics analysis comparing TSS with more vs. less nascent transcription. Finally, we ran motif enrichment positional profiling on these TSS using MEPP.

We conducted the data acquisition in two parts, described below:

1. Fly cell culture and treatment
2. Library prep and csRNA-seq on fly cells

We conducted the data analysis in three parts, also described below:

1. Core promoter element motif library compilation
2. TSS quantification from csRNA-seq
3. MEPP analysis of fly core promoter elements

##### **Fly cell culture and treatment**

Canton S wild-type Drosophila melanogaster flies were grown at 25˚C at 70-80% humidity in population cages. The embryos were collected on molasses-agar plates covered with yeast.

**Library prep and csRNA-seq on fly cells**

CsRNA-seq was performed on embryonic (0-12h) Drosophila melanogaster cells as previously described [(2)](https://paperpile.com/c/mx3Imw/AvFyA). Small RNAs of ~20-60 nt were size selected form 2-5 µg of total RNA by denaturing gel electrophoresis. A 10% input sample was taken aside and the remainder enriched for 5’Caped RNAs with 3’-OH. Monophosphorylated RNAs were selectively degraded by Terminator 5´-Phosphate-Dependent Exonuclease (Lucigen). Subsequent 5’dephosporylation by CIP (NEB) followed by decapping with RppH (NEB) augments Cap-specific 5’adapter ligation by T4 RNA ligase 1 (NEB). The 3’ adapter was ligated using truncated T4 RNA ligase 2 (NEB) without prior 3’ repair to select against degraded RNA fragments.

##### **Core promoter element motif library compilation**

We transformed the Drosophila core promoter motifs from Ohler et al. from log-odds space to probability space [(3)](https://paperpile.com/c/mx3Imw/8mTiQ), then exported the motifs in JASPAR motif format.

##### **TSS quantification from csRNA-seq**

First, 3’ adapters were cut using the the following command:

homerTools trim -3 AGATCGGAAGAGCACACGTCT -mis 2 -minMatchLength 4 -min 20 {reads.fastq.gz}

Where {reads.fastq.gz} stands in for the actual read file.

Then the trimmed reads were aligned to the dm6 reference genome using the STAR aligner, with splice junctions informed by the corresponding Ensemble Genes GTF file available from UCSC [(4, 5)](https://paperpile.com/c/mx3Imw/WdC5i+ubwOl). The read alignments were converted into tag directories using HOMER. A HOMER script, getTSSfromReads.pl, was then used to identify individual TSS from the assay from the 5’ ends of the aligned reads, and score them by their coverage (using “-min 3” to accept only TSS with a minimum count of 3 reads).

The resulting TSS file was converted to a scored bed file using awk, and coverage scores were transformed using the function log2(x+1), where x was the original score. Overlapping interval deduplication was performed for clusters of TSS within 200 bp of each other and on the same strand, selecting the most highly covered TSS within a cluster. Sequence +/- 200 bp of each TSS was extracted from the dm6 reference genome, and scored by csRNA-seq coverage. We proceeded with these scored sequences for downstream analysis with MEPP.

##### **MEPP analysis of fly core promoter elements**

We ran MEPP on the resulting dataset using 200 permutations for profile permutation testing, and filtered out input sequences that were over 50% degenerate. The effective command line invocation was:

mepp --fa {scored_sequences.fa} --motifs {motifs.txt} --out {output_filepath} --orientations +,- --perms 200 --batch 200 --dgt 50 --jobs 20 --gjobs 10 --nogpu --dpi 100 &> {logfile}

#### **MEPP analysis of GATA1 ChIP-seq**

We started by retrieving alignment files for a GATA1 ChIP-seq experiment from K562 cells (ENCODE experiment ENCSR000EFT), as well as corresponding input experiments from ENCODE (ENCODE experiment ENCSR000EHM) [(6, 7)](https://paperpile.com/c/mx3Imw/1vl1s+TaCsF).

In order to obtain differential scoring information, we first derived coverage bigwig files from the alignment files using deeptools bamCoverage defaults [(8)](https://paperpile.com/c/mx3Imw/iysK4). We then used wiggletools to compute two wig files [(9)](https://paperpile.com/c/mx3Imw/MYzhn): one containing the sum of coverage across all replicates, and one containing the log2 fold change of the mean coverage (plus one) between GATA1 ChIP-seq and input control. We calculated the log2 fold change such that higher scores indicated greater sequencing coverage in the GATA1 ChIP-seq experiments. Conversions between wig and bigWig formats were performed using bigWigToWig and wigToBigWig [(10)](https://paperpile.com/c/mx3Imw/ji3EQ).

We then used macs2 callpeak to call peak summits on each bam file [(11)](https://paperpile.com/c/mx3Imw/7tCrf). We concatenated all unique peak summits, finally using bedtools slop to expand +/-100bp from the summits. Using the bedops utilities wig2bed and bedmap [(12)](https://paperpile.com/c/mx3Imw/pxpK2), we annotated these summits with scores from the previously generated log2 fold change bigWig files. We ran the bedmap command in --wmean mode, so that each interval (surrounding a summit) acquired a score equivalent to the mean of overlapping bigWig intervals, weighted by the percentage of overlap. A similar operation assigned a sum of coverage score to each summit from the previously generated bigWig files.

We then performed overlapping interval deduplication of the motif-centered intervals, keeping only the highest-covered interval among clusters of overlapping intervals, and additionally ensuring a minimum total coverage of 5 reads at each interval. Finally, we extracted scored sequences (sampled from the hg38 reference genome) from the deduplicated intervals, and submitted these to MEPP. The effective command line invocation was:

mepp --fa {scored_sequences.fa} --motifs {motifs.txt} --out {output_filepath} --orientations +/- --margin 5 --perms 100 --batch 200 --dgt 50 --jobs 15 --gjobs 15 --nogpu --dpi 100 &> {logfile}

#### **Analysis of differential chromatin accessibility between cell types**

We started by retrieving alignment files for ATAC-seq experiments on K562 and HCT116 cells from ENCODE [(6, 7)](https://paperpile.com/c/mx3Imw/TaCsF+1vl1s). Specifically, we used replicate alignment files from experiment ID ENCSR483RKN on K562 cells, and experiment ID ENCSR872WG on HCT116 cells [(6, 7)](https://paperpile.com/c/mx3Imw/TaCsF+1vl1s).

In order to obtain differential scoring information, we first derived coverage bigwig files from the alignment files, then calculated a log2 fold change bigWig file comparing coverage between HCT116 and K562 cells (using the same methods described above for GATA1 ChIP-seq). We calculated the log2 fold change such that higher scores indicated greater chromatin accessibility in HCT116 cells than in K562 cells. Similarly, we calculated the summed coverage across all samples as a bigWig file.

We then scanned the hg38 human genome for instances of the GATA1 binding motif (as stored in HOMER’s motif library under gata.motif) using the HOMER scanMotifGenomeWide.pl command [(13)](https://paperpile.com/c/mx3Imw/1WLrR). The effective command line was:

scanMotifGenomeWide.pl homer_motifs/gata.motif hg38.fa -bed -5p > gata.scans.bed

This yielded a BED file of genomic intervals +/-100bp centered on the 5’ end of GATA1 binding motifs, and standed according to motif orientation. We annotated these genomic intervals with scores from the log2 fold change bigWig files, as described for the GATA1 ChIP-seq analysis above.. We also repeated this process to annotate the genomic intervals with their overall summed coverage (to support overlapping interval deduplication).

Then as in previous analyses, we performed overlapping interval deduplication of the motif-centered intervals, keeping only the highest-covered interval among clusters of overlapping intervals and additionally ensuring a minimum total coverage of 5 reads at each interval. Finally, we extracted scored sequences (sampled from the hg38 reference genome) from the deduplicated intervals, and submitted these to MEPP. The effective command line invocation was:

mepp --fa {scored_sequences.fa} --motifs {motifs.txt} --out {output_filepath} --orientations +,- --perms 100 --batch 200 --dgt 50 --jobs 20 --gjobs 10 --nogpu --dpi 100 &> {logfile}

#### **Analysis of GATA1 chromatin accessibility with CentriMo, TFEA, and HOMER**

To compare motif analysis methods to MEPP, we analyzed the GATA1 chromatin accessibility data using CentriMo, TFEA, and HOMER, focusing on GATA1 motifs in regions with higher chromatin accessibility in K562 cells. The same HOMER TF motif library was used for each analysis. Because CentriMo and HOMER take contrasting sets of sequences as input, rather than continuously scored sequences, we submitted the lower 10% of scored sequences as the “positive” set for enrichment, and the upper 10% as a contrasting “negative” set. Filenames are simplified for brevity.

HOMER was run with the following command:

findMotifsGenome.pl lower.regions.txt hg38r MotifOutput -bg upper.regions.txt -size 200

CentriMo was run with the following commands:

centrimo --oc lower_vs_upper.centrimo --neg upper.fa --norc --sep --local --noseq lower.fa homer.motifs.id_fixed.meme

TFEA was run with the following command:

TFEA --output ranked_sequences.tfea --fasta_file ranked_sequences_for_tfea.fa --label1 hct116 --label2 k562 --genomefasta hg38.fa --fimo_motifs homer.motifs.id_fixed.meme --fimo_thresh 1e-04 --fimo_background False --output_type 'html' --plotall --cpus 8 --metaprofile --largewindow 100 --smallwindow 10

#### **Analysis of Nanog motif binding in Mus musculus**

First, we scanned the mm10 mouse reference genome for matches to the Nanog binding motif. Using the scanMotifGenomeWide.pl script we obtained a motif scan bed file of intervals +/- 100bp of the motif 5’ end. We reproduce the effective command line below:

scanMotifGenomeWide.pl nanog.motif mm10.masked.fa -bed -5p > mm10.nanog_motif_scans.bed

As a source of Nanog binding signal, we used the normalized Nanog ChIP-seq read signal from GSM4291126, available through GEO Series GSE144577 [(14)](https://paperpile.com/c/mx3Imw/4LN2b). The signal came from a GEO download in the bigWig format, under the file name “GSM4291126_WT_Nanog.bam.bw”. To map this Nanog binding signal data to our motif scans, we first converted the bigWig file to bed file with a combination of bigWigtoWig and wig2bed [(10, 12)](https://paperpile.com/c/mx3Imw/ji3EQ+pxpK2). We then used the bedmap to assign coverage scores from the converted bigWig file to the motif scan bed file, using the ‘wmean’ operation. After filtering out motif scans with zero coverage and performing overlapping interval deduplication, we recovered the sequences (sampled from the mm10 reference genome) within +/- 200bp of the motif 5’ ends. To determine enrichment of motifs in HOMER’s vertebrate TF motif collection, we submitted these scored sequences to MEPP for downstream analysis.

#### **MEPP Analysis of Mouse ChIP-nexus and ChIP-seq**

We started by retrieving MACS2-generated narrowpeak files from GEO accession GSE137193, corresponding to peaks called from ChIP-nexus and ChIP-seq binding assays of transcription factors Nanog, Oct4, and Sox2 on mouse embryonic stem cells [(11, 15)](https://paperpile.com/c/mx3Imw/MwAIB+7tCrf). For each interval in each narrowpeak file, we relocated both start and end positions to the peak summit (using the peak value in column 10), set the score to the signal value (column 7), and the strand to positive, outputting the resulting scored peak summits into a bed file. We then extracted scored sequences +/-200bp of each scored peak summit, and analyzed these scored sequences using MEPP. To account for the less positionally specific nature of ChIP-seq, we used a wider motif margin of 5. To account for the lack of strand specificity in the MACS2 ChIP-seq peak calls, we correlate against the higher motif match score of either motif orientation.

We further demonstrate results from an alternative analysis method for ChIP-seq. Following analysis steps as detailed in GEO accession GSM4072778, we started from the reads for the Nanog ChIP-nexus and patchcap experiments, beginning with barcode trimming via nimnexus trim, including alignment, until aligned read deduplication using nimnexus dedup. We converted the resulting alignments into tag directories. The HOMER getTSSFromReads.pl script was used (in a similar manner to its use in csRNA-seq) To enumerate stranded 1bp binding sites from the 5’ ends of each tag in these tag directories we used the HOMER getTSSFromReads.pl script, ensuring at least 4 reads were counted per site (-minRaw 4), while controlling for 5’ ends from the patchcap experiment. We then used the HOMER annotatePeaks.pl command to count 5’ ends of strand-matched tags from both tag directories at each enumerated site, as well as to normalize these counts using DESeq2’s rlog method. The resulting 1bp binding sites were scored by the rlog-normalized coverage of tag 5’ ends from the Nanog ChIP-nexus experiment, expanded by 200bp in each direction then cluster deduplicated (favoring sites with higher coverage). Finally, we converted the scored and expanded binding sites into scored sequences (sampled from the mm10 reference genome), which we analyzed using MEPP, carrying over parameters from the previous ChIP-nexus analysis.

#### **Differential csRNA-seq analysis**

csRNA-seq data from mouse bone marrow derived macrophage cells treated with KLA was downloaded from GSE135498 [(2)](https://paperpile.com/c/mx3Imw/AvFyA). Subsequently, we ran TSS identification and differential TSS analysis between the KLA-stimulated and control samples.

The data analysis was conducted in three parts, described below:

1. TSS quantification from csRNA-seq on mouse BMDM cells
2. Differential TSS analysis and scoring
3. MEPP analysis of differential TSS

##### **TSS quantification from csRNA-seq on mouse BMDM cells**

TSS quantification proceeded as described above for fly cells, with the exception that alignment was to mm10 references, and with a minimum read count of 5 (-minRaw 5). Using HOMER’s annotatePeaks.pl command, we then quantified 5’ end read counts. We reproduce the effective command line below:
 annotatePeaks.pl tss.txt genomes/mm10/mm10.fa -strand + -fragLength 1 -raw -d mm10_csrna_raw_kla_0min_replicate_1/ mm10_csrna_raw_kla_0min_replicate_2/ mm10_csrna_raw_kla_50min_replicate_1/ mm10_csrna_raw_kla_50min_replicate_2/ > tss.counts.txt

##### **Differential TSS analysis and scoring**

We performed differential csRNA-seq analysis on the previously generated TSS read count quantifications using HOMER’s getDiffExpression.pl script, (a wrapper around DESeq2) [(16)](https://paperpile.com/c/mx3Imw/1HG1D). The comparison found log2 fold change values comparing TSS transcriptional signal in KLA-stimulated vs. control, after controlling for replicate batch effects.

##### **MEPP analysis of differential TSS**

We scored TSS by their log2 fold change values, then performed overlapping interval deduplication, selecting the TSS from each cluster with the highest read coverage. We then extracted sequence +/- 200bp around the TSS, and input the resulting scored sequences into MEPP, to analyze enrichment of motifs in HOMER’s vertebrate TF motif collection.

#### **Differential cleavage site analysis**

We started by retrieving ATAC-seq and H3K27ac MNase-seq data for BMDM cells treated to 1 hour of LPS stimulation vs. control, from the work of Comoglio et al., available under GEO Series GSE119693 [(17)](https://paperpile.com/c/mx3Imw/8WSYu). We then trimmed adapters from reads using Trim Galore, then aligned the reads to the mm10 reference genome using bowtie2 [(18, 19)](https://paperpile.com/c/mx3Imw/FsF6z+HfXXZ). After converting the read alignments to HOMER tag directories, the rest of the analysis proceeded as described above for differential csRNA-seq analysis.

## **Supplementary Methods References**

[1. Quinlan,A.R. (2014) BEDTools: The Swiss‐Army Tool for Genome Feature Analysis. *Current Protocols in Bioinformatics*, **47**.](http://paperpile.com/b/mx3Imw/u8Lp)

[2. Duttke,S.H., Chang,M.W., Heinz,S. and Benner,C. (2019) Identification and dynamic quantification of regulatory elements using total RNA. *Genome Res.*, **29**, 1836–1846.](http://paperpile.com/b/mx3Imw/AvFyA)

[3. Ohler,U., Liao,G.-C., Niemann,H. and Rubin,G.M. (2002) Computational analysis of core promoters in the Drosophila genome. *Genome Biol.*, **3**, RESEARCH0087.](http://paperpile.com/b/mx3Imw/8mTiQ)

[4. Dobin,A., Davis,C.A., Schlesinger,F., Drenkow,J., Zaleski,C., Jha,S., Batut,P., Chaisson,M. and Gingeras,T.R. (2013) STAR: ultrafast universal RNA-seq aligner. *Bioinformatics*, **29**, 15–21.](http://paperpile.com/b/mx3Imw/WdC5i)

[5. Howe,K.L., Achuthan,P., Allen,J., Allen,J., Alvarez-Jarreta,J., Amode,M.R., Armean,I.M., Azov,A.G., Bennett,R., Bhai,J., *et al.* (2021) Ensembl 2021. *Nucleic Acids Res.*, **49**, D884–D891.](http://paperpile.com/b/mx3Imw/ubwOl)

[6. Davis,C.A., Hitz,B.C., Sloan,C.A., Chan,E.T., Davidson,J.M., Gabdank,I., Hilton,J.A., Jain,K., Baymuradov,U.K., Narayanan,A.K., *et al.* (2018) The Encyclopedia of DNA elements (ENCODE): data portal update. *Nucleic Acids Res.*, **46**, D794–D801.](http://paperpile.com/b/mx3Imw/1vl1s)

[7. ENCODE Project Consortium (2012) An integrated encyclopedia of DNA elements in the human genome. *Nature*, **489**, 57–74.](http://paperpile.com/b/mx3Imw/TaCsF)

[8. Ramírez,F., Dündar,F., Diehl,S., Grüning,B.A. and Manke,T. (2014) deepTools: a flexible platform for exploring deep-sequencing data. *Nucleic Acids Res.*, **42**, W187–91.](http://paperpile.com/b/mx3Imw/iysK4)

[9. Zerbino,D.R., Johnson,N., Juettemann,T., Wilder,S.P. and Flicek,P. (2014) WiggleTools: parallel processing of large collections of genome-wide datasets for visualization and statistical analysis. *Bioinformatics*, **30**, 1008–1009.](http://paperpile.com/b/mx3Imw/MYzhn)

[10. Kent,W.J., Zweig,A.S., Barber,G., Hinrichs,A.S. and Karolchik,D. (2010) BigWig and BigBed: enabling browsing of large distributed datasets. *Bioinformatics*, **26**, 2204–2207.](http://paperpile.com/b/mx3Imw/ji3EQ)

[11. Feng,J., Liu,T., Qin,B., Zhang,Y. and Liu,X.S. (2012) Identifying ChIP-seq enrichment using MACS. *Nat. Protoc.*, **7**, 1728–1740.](http://paperpile.com/b/mx3Imw/7tCrf)

[12. Neph,S., Kuehn,M.S., Reynolds,A.P., Haugen,E., Thurman,R.E., Johnson,A.K., Rynes,E., Maurano,M.T., Vierstra,J., Thomas,S., *et al.* (2012) BEDOPS: high-performance genomic feature operations. *Bioinformatics*, **28**, 1919–1920.](http://paperpile.com/b/mx3Imw/pxpK2)

[13. Heinz,S., Benner,C., Spann,N., Bertolino,E., Lin,Y.C., Laslo,P., Cheng,J.X., Murre,C., Singh,H. and Glass,C.K. (2010) Simple combinations of lineage-determining transcription factors prime cis-regulatory elements required for macrophage and B cell identities. *Mol. Cell*, **38**, 576–589.](http://paperpile.com/b/mx3Imw/1WLrR)

[14. Sun,X., Ren,Z., Cun,Y., Zhao,C., Huang,X., Zhou,J., Hu,R., Su,X., Ji,L., Li,P., *et al.* (2020) Hippo-YAP signaling controls lineage differentiation of mouse embryonic stem cells through modulating the formation of super-enhancers. *Nucleic Acids Res.*, **48**, 7182–7196.](http://paperpile.com/b/mx3Imw/4LN2b)

[15. Avsec,Ž., Weilert,M., Shrikumar,A., Krueger,S., Alexandari,A., Dalal,K., Fropf,R., McAnany,C., Gagneur,J., Kundaje,A., *et al.* (2021) Base-resolution models of transcription-factor binding reveal soft motif syntax. *Nat. Genet.*, **53**, 354–366.](http://paperpile.com/b/mx3Imw/MwAIB)

[16. Love,M.I., Huber,W. and Anders,S. (2014) Moderated estimation of fold change and dispersion for RNA-seq data with DESeq2. *Genome Biol.*, **15**, 550.](http://paperpile.com/b/mx3Imw/1HG1D)

[17. Comoglio,F., Simonatto,M., Polletti,S., Liu,X., Smale,S.T., Barozzi,I. and Natoli,G. (2019) Dissection of acute stimulus-inducible nucleosome remodeling in mammalian cells. *Genes Dev.*, **33**, 1159–1174.](http://paperpile.com/b/mx3Imw/8WSYu)

[18. Krueger,F., James,F., Ewels,P., Afyounian,E. and Schuster-Boeckler,B. (2021) FelixKrueger/TrimGalore: v0.6.7 - DOI via Zenodo Zenodo.](http://paperpile.com/b/mx3Imw/FsF6z)

[19. Langmead,B. and Salzberg,S.L. (2012) Fast gapped-read alignment with Bowtie 2. *Nat. Methods*, **9**, 357–359.](http://paperpile.com/b/mx3Imw/HfXXZ)

## **Supplemental Figures & Tables**

#####


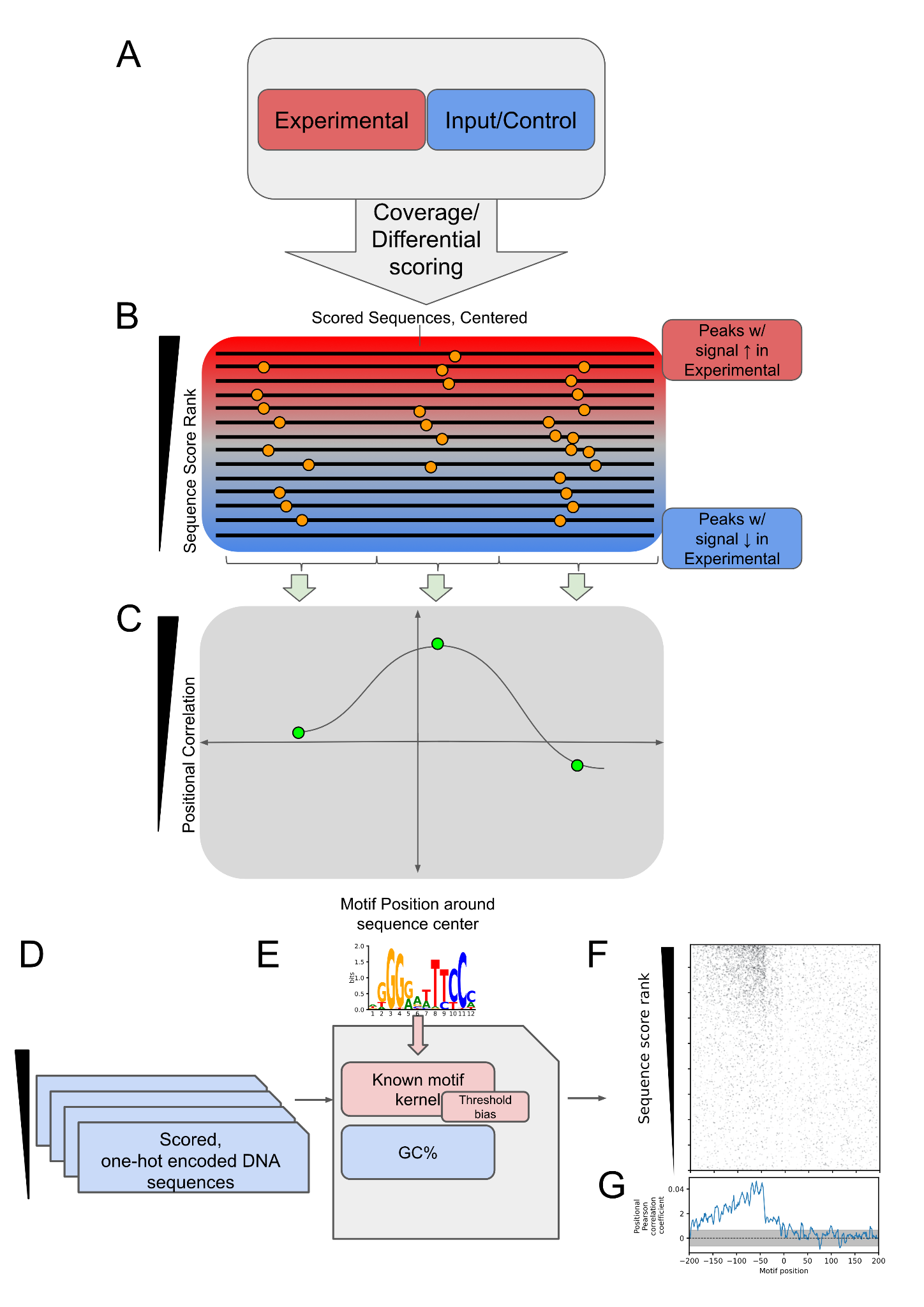


##### **Supplemental Figure S1: MEPP uses pre-weighted convolutional kernels to derive motif heatmaps and positional correlation profiles**

1. Typical differential sequencing experiment with replicates, yielding peaks scored by a function of coverage (e.g. normalized or log 2 fold change coverage).
2. Scored sequences are extracted from scored peaks, centered on some feature of interest, e.g. TSS. Motif locations marked in orange.
3. Local motif enrichments yield positional correlations of the motif with sequence score.
4. Input to MEPP consists of a set of scored, one-hot encoded DNA sequences
5. A known motif is input to form the weights of a convolutional kernel, which evaluates the log odds matching score of a one-hot encoded sequence to the motif. The detection threshold for the motif inverted to form the kernel’s bias. The resulting activation value is fed through a ReLU function. The motif’s GC% is also evaluated.
6. The entire set of scored sequences is convolved, forming rows of a motif heatmap. Rows are sorted in descending order from the score of the corresponding input sequence.
7. Local correlations of motif match scores and sequence scores are calculated for each motif position, controlling for GC% skew using a partial Pearson correlation.


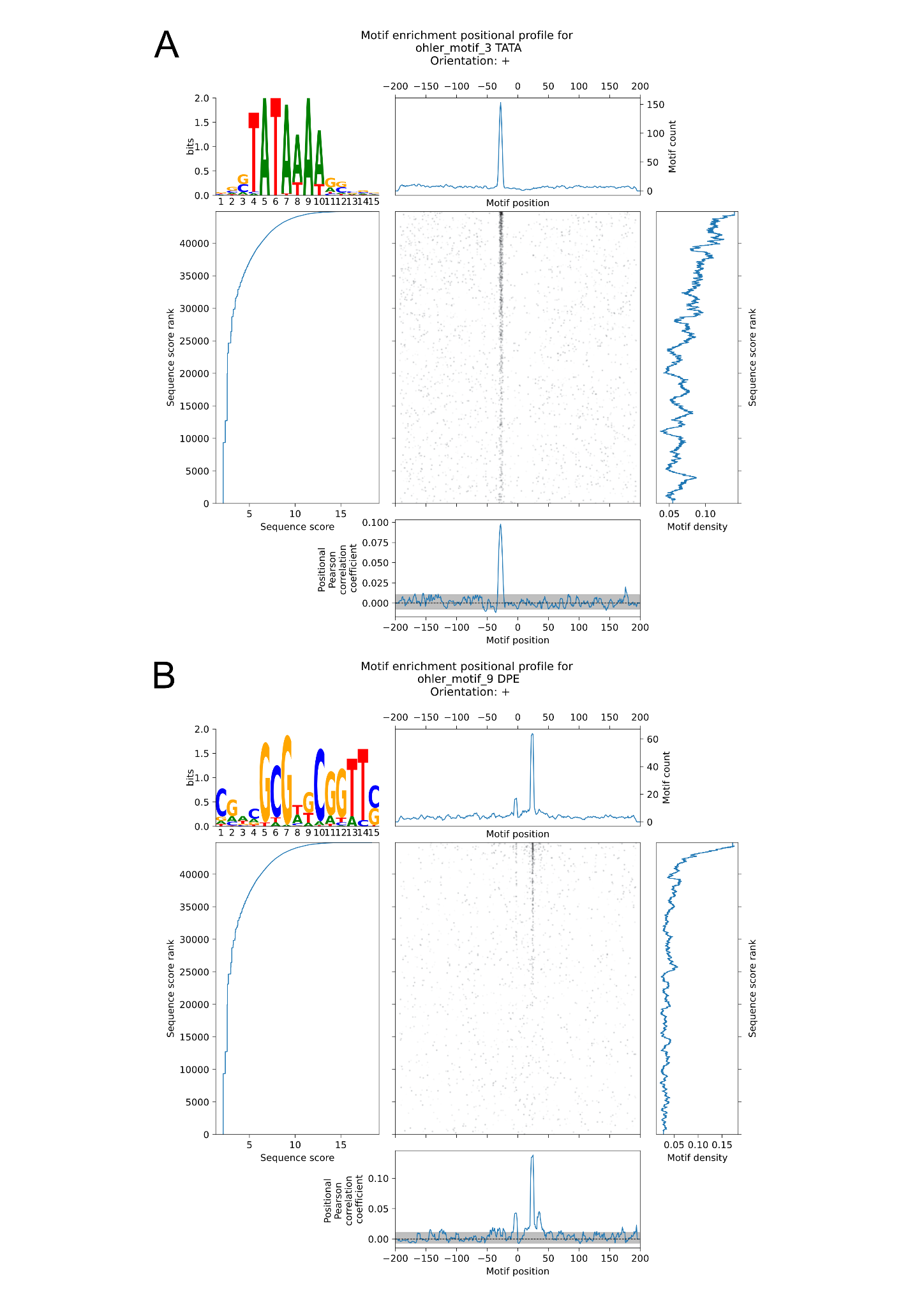


##### **Supplemental Figure S2: MEPP visualizes and quantifies the TATA-box and DPR motifs near Drosophila melanogaster transcription start sites.**

1. MEPP plot for TATA-box motif (from the HOMER motif collection), on sequences +/- 200bp of *D. melanogaster* TSS quantified by csRNA-seq, scored by log-transformed csRNA-seq coverage.
2. Same as (A) but for the DPR motif (from the HOMER motif collection)

#####
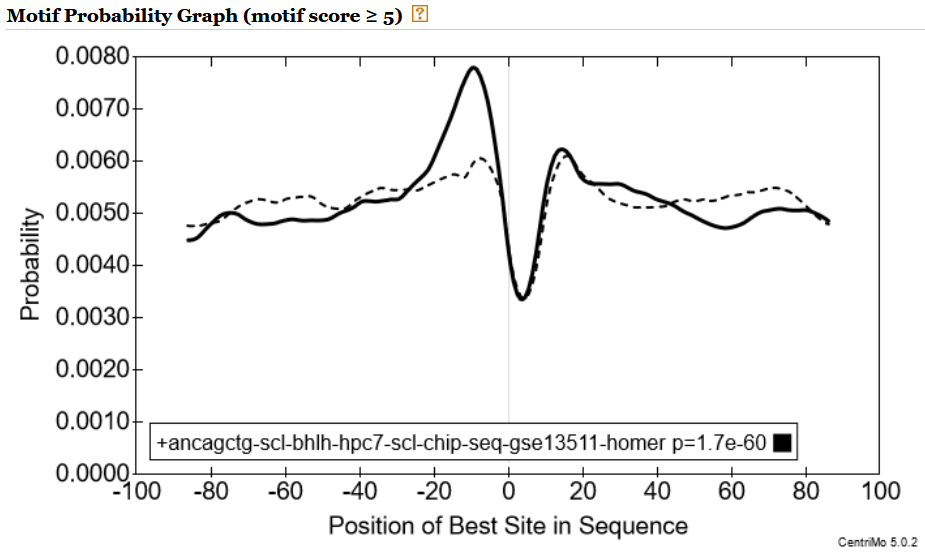


##### **Supplemental Figure S3: CentriMo visualizes motif prevalence over sequence positions**

Local enrichment plot for SCL binding motif locations surrounding GATA1 binding motifs as generated by CentriMo, using default visualization smoothing parameters. Enrichment was performed contrasting the lower 10% of scored sequences with the upper 10% of scored sequences.

#####


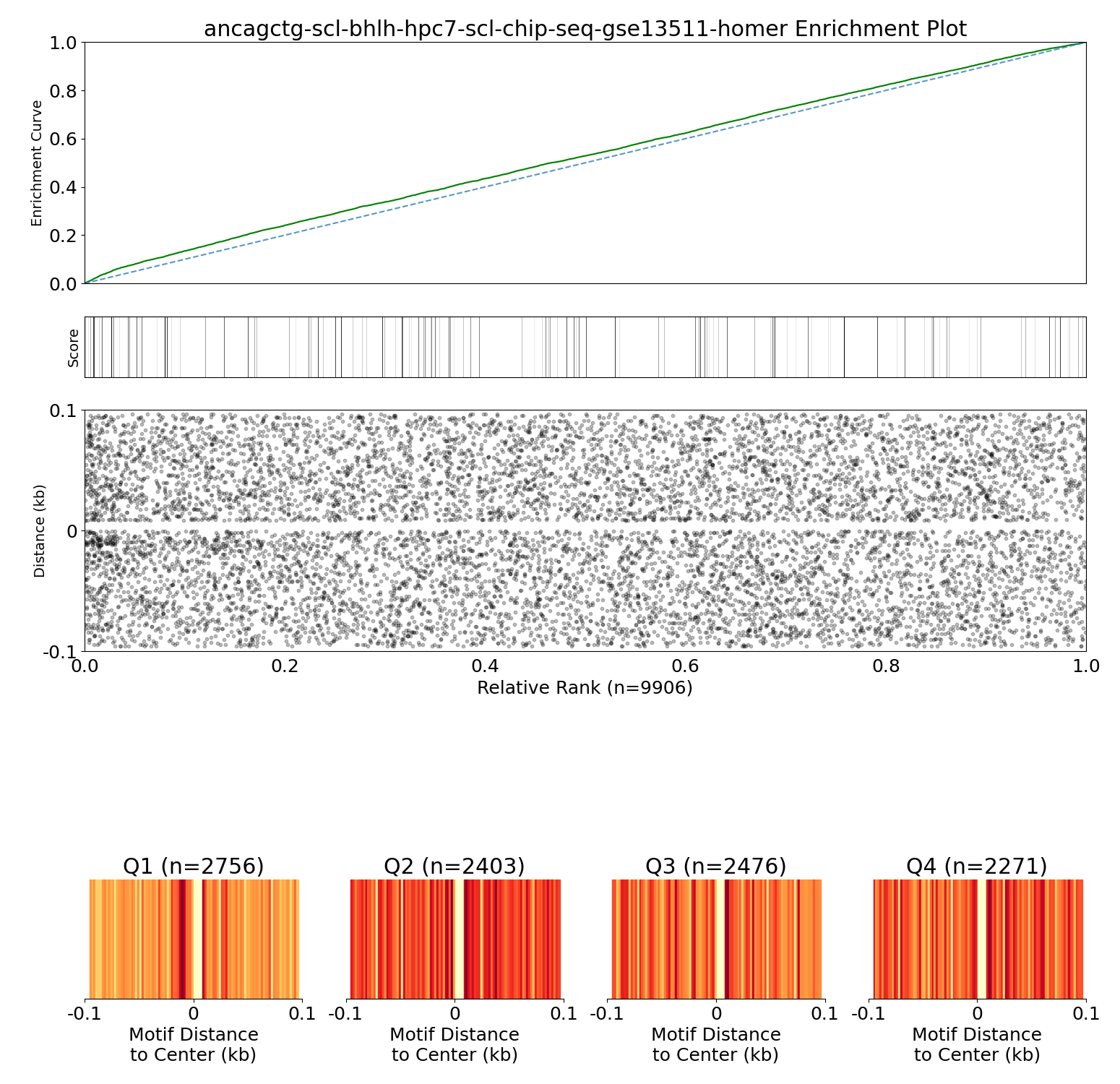


##### **Supplemental Figure S4: TFEA visualizes motif positions and motif prevalence in quartiles of the data**

Enrichment plot generated by TFEA for SCL binding motif locations surrounding GATA1 binding motifs. The plot includes heatmaps describing positional motif counts in quartiles of the data. Q1 indicates the regions that are most specific to K562 cells, where SCL motifs are positionally enriched just upstream of the central GATA1 motif.

##### **Supplemental Table S5: Top motif families found by MEPP, TFEA, HOMER, and CentriMo**

| **Method** | **Top Result Rank** | **Motif Name** | **Enrichment Metrics** | | |
| --- | --- | --- | --- | --- | --- |
| **MEPP** | **MEPP Results** | | **abs_extreme_r** | **extreme_r** | **extreme_r_pos** |
|  | 1 | GATA:SCL(Zf,bHLH)^1^ | 0.101083 | -0.101083 | -6 |
|  | 2 | Gata1(Zf)^2^ | 0.042719 | -0.042719 | 2 |
|  | 4 | Ascl2(bHLH)^3^ | 0.022561 | -0.022561 | -12 |
|  | 12 | Fra2(bZIP) | 0.018521 | 0.018521 | 93 |
| **TFEA (positive e-score table)** | **TFEA Results** | | **E-Score** | **Corrected E-Score** | |
|  | 1 | GATA:SCL(Zf,bHLH)^1^ | 0.12 | 0.118 | |
|  | 2 | Gata6(Zf)^2^ | 0.0181 | 0.0243 | |
|  | 8 | Elf4(ETS) | 0.075 | 0.0728 | |
|  | 10 | MyoD(bHLH)^3^ | 0.0604 | 0.0536 | |
|  | 15 | STAT5(Stat) | 0.0545 | 0.0618 | |
| **HOMER** | **HOMER Results** | | **Log P-value** | | |
|  | 1 | GATA:SCL(Zf,bHLH)^1^ | -9.24E+02 | | |
|  | 2 | SpiB(ETS) | -7.56E+01 | | |
|  | 3 | WT1(Zf) | -7.30E+01 | | |
|  | 5 | BMYB(HTH) | -6.05E+01 | | |
|  | 6 | STAT5(Stat) | -4.99E+01 | | |
|  | 13 | Twist2(bHLH)^3^ | -4.02E+01 | | |
|  | 22 | Tgif1(Homeobox) | -3.55E+01 | | |
| **CentriMo (lower vs. upper)** | **CentriMo Results** | | **Fisher E-value** | | |
|  | 1 | Olig2(bHLH)^3^ | 4.5E-29 | | |
|  | 20 | ZBTB18(Zf) | 2.1E-14 | | |

##### **Legend & Color Coding for Table S5**

Each motif analysis method was used to analyze regions near GATA1 motifs that were specifically enriched for accessible chromatin in K562 cells compared to HCT116 cells. The top results returned by each method are listed in the table, keeping only the top result for motifs with the same DNA binding domain to remove redundancy, and stopping after the first novel family after bHLH. The top reported instances of bHLH motifs, which can be found with a specific spacing to GATA1 motifs at GATA1 binding sites in erythroid lineage cells, are highlighted in yellow. Results recognizing GATA or the composite GATA+bHLH (true positive) are highlighted in green and blue, respectively.

1. Blue - GATA:SCL (Zf,bHLH) composite motif
2. Green - GATA family (Zf) motif
3. Yellow - E-box (bHLH) motif

##### **Supplemental Table S6: Public Data accessions and attributions used in this study**

| **Dataset accession (s)** | **Sample/File accessions** | **Sample Title** | **Producing Lab** |
| --- | --- | --- | --- |
| ENCSR000EFT | ENCFF844WTT.bam | GATA1 ChIP-seq rep 1 | Michael Snyder, Stanford |
| ENCSR000EFT | ENCFF729MTL.bam | GATA1 ChIP-seq rep 2 |  |
| ENCSR000EHM | ENCFF769RAH.bam | Control ChIP-seq rep 1 |  |
| ENCSR000EHM | ENCFF147YPF.bam | Control ChIP-seq rep 2 |  |
| ENCSR483RKN | ENCFF512VEZ.bam | K562 ATAC-seq rep 1 |  |
| ENCSR483RKN | ENCFF987XOV.bam | K562 ATAC-seq rep 2 |  |
| ENCSR872WG | ENCFF724QHH.bam | HCT116 ATAC-seq rep 1 |  |
| ENCSR872WG | ENCFF927YUB.bam | HCT116 ATAC-seq rep 2 |  |
| GSE144577 | GSM4291126_WT_Nanog.bam.bw | WT_Nanog_ChIP | Xiang Sun, Sun Yat-sen University |
| GSE137193 | GSM4087827_mesc_Nanog_chipseq.idr-optimal-set.narrowPeak.gz | Nanog-ChIP-seq | Julia Zeitlinger, Stowers Institute for Medical Research |
| GSE137193 | GSM4072778_mesc_nanog_nexus.idr-optimal-set.narrowPeak.gz | Nanog-ChIP-nexus |  |
| GSE137193 | SRX6827272 | Nanog-ChIP-nexus |  |
| GSE135498 | GSM4012734 | BMDM notx csRNA-seq r1 | Christopher Benner, UCSD |
| GSE135498 | GSM4012735 | BMDM notx csRNA-seq r2 |  |
| GSE135498 | GSM4012736 | BMDM notx csRNAinput r1 |  |
| GSE135498 | GSM4012737 | BMDM notx csRNAinput r2 |  |
| GSE135498 | GSM4012738 | BMDM KLA1h csRNA-seq r1 |  |
| GSE135498 | GSM4012739 | BMDM KLA1h csRNA-seq r2 |  |
| GSE135498 | GSM4012740 | BMDM KLA1h csRNAinput r1 |  |
| GSE135498 | GSM4012741 | BMDM KLA1h csRNAinput r2 |  |
| GSE119693 | GSM3380863 | ATAC_LPS-1h_rep1 | Gioacchino Natoli, Humanitas University (Hunimed) |
| GSE119693 | GSM3380864 | ATAC_LPS-1h_rep2 |  |
| GSE119693 | GSM3380865 | ATAC_LPS-1h_rep3 |  |
| GSE119693 | GSM3380875 | ATAC_UT_rep1 |  |
| GSE119693 | GSM3380876 | ATAC_UT_rep2 |  |
| GSE119693 | GSM3380877 | ATAC_UT_rep3 |  |
| GSE119693 | GSM3380878 | H3K27ac_LPS-1h_rep1 |  |
| GSE119693 | GSM3380879 | H3K27ac_LPS-1h_rep2 |  |
| GSE119693 | GSM3380886 | H3K27ac_UT_rep1 |  |
| GSE119693 | GSM3380887 | H3K27ac_UT_rep2 |  |
